# *N*-acetylglucosamine utilization and impact on antibiotic susceptibility, oxidative stress tolerance, and swimming in *Stenotrophomonas maltophilia*

**DOI:** 10.1128/spectrum.03167-25

**Published:** 2026-03-16

**Authors:** Chun-Hsing Liao, Hsu-Feng Lu, Shao-Chi Wu, En-Wei Hu, Li-Hua Li, Yi-Tsung Lin, Tsuey-Ching Yang

**Affiliations:** 1 Division of Infectious Disease, Far Eastern Memorial Hospital 46608, New Taipei City, Taiwan; 2 Department of Medicine, National Yang Ming Chiao Tung University 34914, Taipei, Taiwan; 3 Department of Medical Laboratory Science and Biotechnology, Asia University 63267, Taichung, Taiwan; 4 Department of Biotechnology and Laboratory Science in Medicine, National Yang Ming Chiao Tung University 34914, Taipei, Taiwan; 5 Department of Pathology and Laboratory Medicine, Taipei Veterans General Hospital 46615, Taipei, Taiwan; 6 School of Medical Laboratory Science and Biotechnology, College of Medical Science and Technology, Taipei Medical University 38032, Taipei, Taiwan; 7 Division of Infectious Diseases, Department of Medicine, Taipei Veterans General Hospital 46615, Taipei, Taiwan; University of Manitoba, Winnipeg, Canada

**Keywords:** *Stenotrophomonas maltophilia*, *N*-acetylglucosamine, swimming, oxidative stress tolerance, antibiotic susceptibility

## Abstract

*N*-acetylglucosamine (GlcNAc) is an amino sugar that serves as a structural component, nutrient source, and signal molecule in bacteria. In this study, we sought to identify the genes involved in GlcNAc utilization in *Stenotrophomonas maltophilia* and to evaluate the impact of GlcNAc utilization on antibiotic susceptibility, oxidative stress tolerance, and swimming motility. The roles of the *nagPIBAF* operon, *nagK*, and *nagA2* in GlcNAc utilization were investigated through mutant construction and growth assays. NagPsm and NagF mediated GlcNAc transport across the inner membrane, while NagK phosphorylated GlcNAc in the cytoplasm. NagA and NagA2 exhibited functional redundancy as *N*-acetylglucosamine-6-phosphate deacetylases, and NagB functioned as a glucosamine-6-phosphate deaminase. The *nagPIBAF* operon was repressed by NagI and derepressed in response to GlcNAc-6P. Antibiotic susceptibility and oxidative stress tolerance were assessed by E-test and menadione IC_50_, respectively. Among the clinical isolates tested, 71.4% (5/7) and 57.1% (4/7) of the strains showed increases in MIC values for colistin and ceftazidime (CAZ) of 1.3 to 2.6 times and 1.3 to 2 times, respectively. Furthermore, GlcNAc utilization enhanced swimming motility but had little effect on oxidative stress tolerance. Overall, the *nagPIBAF* operon, *nagK*, and *nagA2* contribute to GlcNAc utilization, which in turn can increase resistance to CAZ and colistin and promote swimming motility in some clinical isolates of *S. maltophilia*.

**IMPORTANCE:**

*N*-acetylglucosamine (GlcNAc) is widely used as a dietary supplement due to its proposed cartilage-protective and anti-inflammatory properties. In bacteria, however, GlcNAc functions as a structural component of peptidoglycan and lipopolysaccharide, as a signal molecule, and as a nutrient source. *Stenotrophomonas maltophilia* is a gram-negative opportunistic pathogen associated with nosocomial infections, particularly in cystic fibrosis (CF) patients. The abundance of amino sugars derived from mucin degradation is present in the CF lung. Utilization of GlcNAc can reprogram bacterial metabolism, leading to pleiotropic effects on physiology and stress tolerance. We were therefore interested in how bacteria adapt their physiology and stress tolerance when residing in GlcNAc-rich infection niches. Here, we investigated GlcNAc utilization and its impact on physiology and stress tolerance in *S. maltophilia*.

## INTRODUCTION

**Fig 1:**
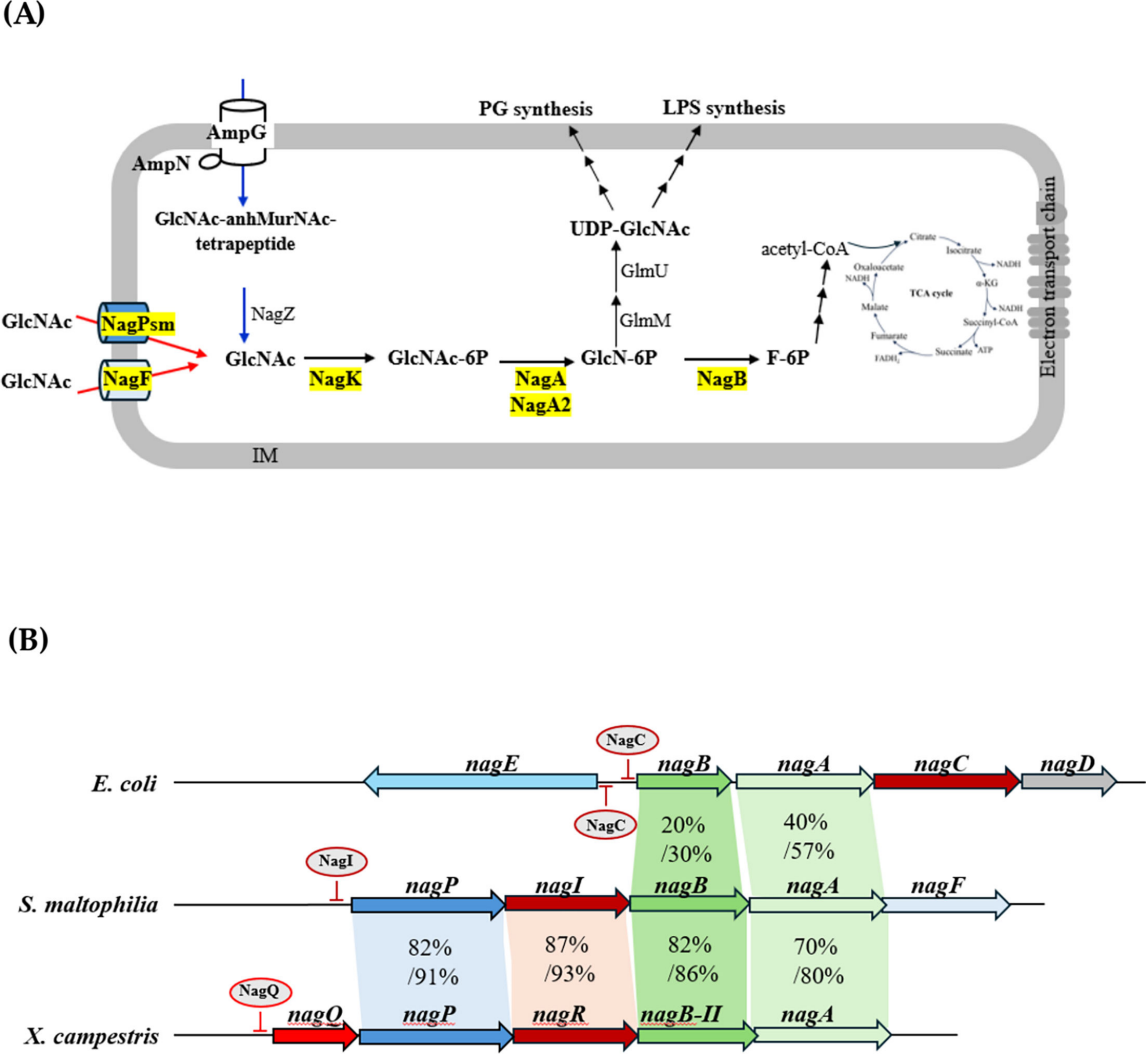
GlcNAc utilization model and genomic organization of GlcNAc utilization-associated genes. (**A**) Proposed GlcNAc utilization pathway in *Stenotrophomonas maltophilia*. GlcNAc can be produced endogenously via PG recycling through the AmpNG permease system (blue) or imported from the extracellular environment (red). Proteins characterized in this study are highlighted in yellow. GlcNAc is transported into the cytoplasm via NagPsm and NagF, phosphorylated by NagK, and deacetylated by NagA and NagA2 to form GlcN-6P, which is either catabolized via NagB or used for UDP-GlcNAc synthesis via GlmM-GlmU system. (**B**) Comparison of GlcNAc utilization genes in *S. maltophilia* with homologs in *Escherichia coli* and *Xanthomonas campestris*. Genes encoding proteins of similar function are colored identically. Numbers in the shaded areas indicate protein identity and similarity between compared proteins.

Amino sugars play dual roles in bacteria: they are structural components of peptidoglycan (PG) and lipopolysaccharide (LPS) in gram-negative bacteria, and they also serve as sources of carbon, nitrogen, and energy. *N*-acetylglucosamine (GlcNAc) is the most common amino sugar in the environment and is a preferred substrate within the hierarchy of catabolite repression (1). For a bacterium, GlcNAc originates either endogenously from PG recycling (blue arrows in Fig. 1A) or exogenously from the extracellular milieu (red arrows in Fig. 1A). PG is a polymer of β−1,4-linked GlcNAc and *N*-acetylmuramic acid (MurNAc) cross-linked by short peptides (2). During PG turnover, the periplasmic GlcNAc-β-anhMurNAc-tetrapeptide is transported into the cytoplasm and hydrolyzed by *N*-acetyl-β-glucosaminidase (NagZ) to release GlcNAc (3) (blue arrows in Fig. 1A).

Exogenous GlcNAc may cross the outer membrane by passive diffusion (4), while inner-membrane transport mechanisms vary among species. In *Escherichia coli*, GlcNAc is imported and phosphorylated concomitantly by the phosphoenolpyruvate-dependent phosphotransferase system (PTS) (5). In contrast, *Xanthomonas campestris* uses NagP, a member of the major facilitator superfamily (MFS), for inner-membrane uptake (4), and *Streptomyces olivaceoviridis* employs the NgcEFG ATP-binding cassette (ABC) transporter (6). When transported by non-PTS systems such as NagP or NgcEFG, GlcNAc is imported in an unphosphorylated form and is subsequently phosphorylated in the cytoplasm by a GlcNAc kinase, which relies on ATP-dependent phosphorylation (4, 6). GlcNAc-6-phosphate (GlcNAc-6P) is then deacetylated by *N*-acetylglucosamine-6-phosphate deacetylase (NagA) to yield GlcN-6P. GlcN-6P can be catabolized toward glycolysis via glucosamine-6-phosphate deaminase (NagB) or diverted into anabolic pathways that regenerate UDP-GlcNAc through the GlmM-GlmU system (7). UDP-GlcNAc is a key precursor for PG and LPS biosynthesis (Fig. 1A). Genes involved in GlcNAc uptake, catabolism, and regulation are often clustered in operons or genomic islands, for example, the *nagE-nagBACD* cluster in *E. coli* (Fig. 1B) (8), the *nagQ-nagP-nagR-nagB-II-nagA* operon in *X. campestris* (Fig. 1B) (4), and the *nagRFE-nagABDC* cluster in *Caulobacter crescentus* (9).

Bacteria routinely encounter fluctuating environments, so rapid and precise transcriptional regulation is essential for survival. DNA-binding transcription factors integrate internal and external signals through allosteric ligand binding, which modulates their affinity for operator DNA and thereby alters downstream gene expression (10). Most transcriptional regulators comprise two domains: an N-terminal helix-turn-helix (HTH) domain for DNA recognition and a C-terminal ligand-binding domain that senses specific effectors—often metabolites or small molecules. Regulators are grouped into families according to sequence similarity in their DNA-binding domains, such as the LysR, MarR, and LacI families. Members of the LacI family commonly regulate carbohydrate utilization: they possess an N-terminal HTH DNA-binding domain and a C-terminal sugar-binding domain, and most act locally as negative regulators of adjacent genes involved in carbon source metabolism (11, 12).

*Stenotrophomonas maltophilia* is a gram-negative nonfermenting bacterium widely distributed in soil, plants, animals, and aqueous environments and is increasingly recognized as an opportunistic nosocomial pathogen, especially in immunocompromised patients and elderly people (13). *S. maltophilia* infections are challenging to treat due to the bacterium’s high intrinsic resistance to common antibiotics. In our recent work, we observed upregulation of the *nagPIBAF* operon in an *ompA* mutant, and this upregulation correlated with reduced oxidative stress tolerance (14). The *nag* locus encodes two predicted inner-membrane permeases (NagPsm and NagF), a LacI-type transcriptional regulator (NagI), and enzymes implicated in GlcNAc metabolism such as NagB (glucosamine-6-phosphate deaminase) and NagA (N-acetylglucosamine-6-phosphate deacetylase) (14). In the present study, we re-examined the *nagPIBAF* operon to define the contribution of its components to GlcNAc utilization and to assess how GlcNAc metabolism affects antibiotic susceptibility, oxidative stress tolerance, and swimming motility in *S. maltophilia*.

## RESULTS

### *Nag* operon contributes to GlcNAc utilization in *S. maltophilia*

**Fig 2:**
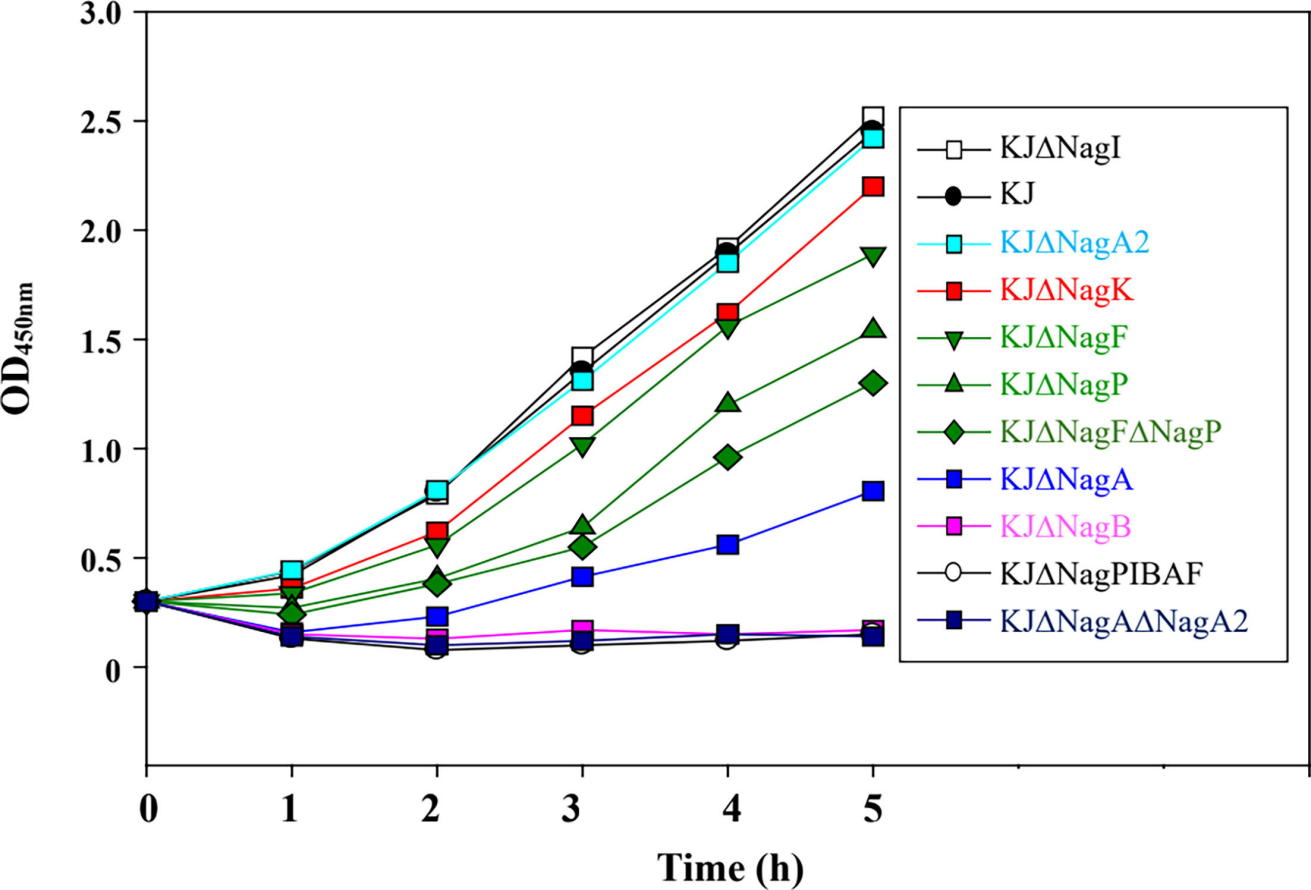
Contribution of *nagPIBAF*, *nagK*, and *nagA2* to growth on GlcNAc as sole carbon source. Overnight cultures were inoculated into XOLN medium with 100 mM GlcNAc at an initial OD_450nm_ of 0.15; growth was followed by OD_450nm_ readings. The graph is representative of at least three independent experiments.

We previously reported that the *nagPIBAF* operon contributes to GlcNAc utilization (14). To dissect the roles of individual genes within this operon, we constructed five single in-frame deletion mutants—KJΔNagP, KJΔNagI, KJΔNagB, KJΔNagA, and KJΔNagF—and one double deletion mutant, KJΔNagPΔNagF. qRT-PCR and the following complementary assay were used to check for polar effects in these mutants. The results of qRT-PCR demonstrated that no polar effects were detected in KJΔNagP, KJΔNagB, and KJΔNagA (Fig. S1). Consistent with our earlier findings, the full *nagPIBAF* deletion (KJΔNagPIBAF) lost viability when cultured in minimal XOLN medium containing GlcNAc as the sole carbon source (Fig. 2). Compared with wild-type KJ, KJΔNagP and KJΔNagF showed impaired growth in XOLN-GlcNAc, and the growth defect was aggravated in the double mutant KJΔNagPΔNagF (Fig. 2), supporting roles for NagPsm and NagF in GlcNAc uptake. *nagPsm* encodes an MFS inner membrane transporter, which displayed 82% identity with *X. campestris* NagP. NagF shares 35% identity with *Tannerella forsythia* MurT, a MurNAc permease (15). KJΔNagB completely lost the ability to grow on XOLN-GlcNAc (Fig. 2). However, KJΔNagA partially retained the ability to grow on XOLN-GlcNAc, albeit at a reduced level relative to wild type (Fig. 2). The mutants that displayed compromised growth were subjected to a complementation assay. The growth of complementary strains was reverted to wild-type level, further verifying that no significant polar effects occur in these deletion mutants assayed. (Fig. S2).

### NagA2 (Smlt4438) contributes to GlcNAc utilization

The essential roles of NagA and NagB for GlcNAc utilization have been reported in other bacteria (4, 8, 9); nevertheless, KJΔNagA still kept partial ability to grow on XOLN-GlcNAc (Fig. 2). This observation suggested functional redundancy for NagA activity in *S. maltophilia*. A genome survey in strain *S. maltophilia* K279a using NagA as a query revealed a homolog, *smlt4438*, encoding a 375-amino acid cytoplasmic protein sharing 50% similarity and 37% identity with NagA; we designated this gene *nagA2*. To probe its role, we constructed KJΔNagA2 and the double mutant KJΔNagAΔNagA2. KJΔNagA2 grew comparably to wild-type KJ on XOLN-GlcNAc, whereas the double mutant KJΔNagAΔNagA2 lost viability in XOLN-GlcNAc (Fig. 2), supporting functional redundancy between NagA and NagA2. To further confirm the contribution of NagA2 to GlcNAc utilization, *nagA* and *nagA2*, individually or together, were transported to KJΔNagAΔNagA2 for a complementary assay. Individual complementation of *nagA* or *nagA2* partially restored the growth of KJΔNagAΔNagA2 in XOLN-GlcNAc, and simultaneous complementation of both genes restored the growth more significantly (Fig. S3).

### NagK (Smlt4435) contributes to GlcNAc phosphorylation in the cytoplasm

**Fig 3:**
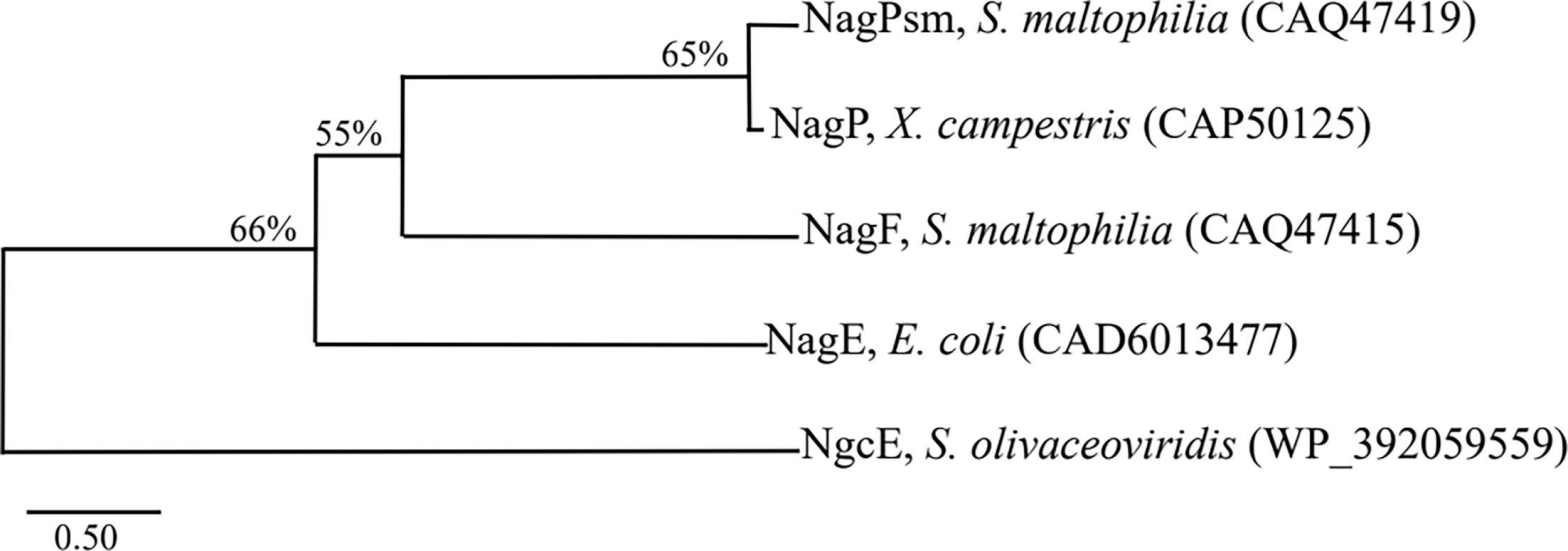
Phylogenetic analysis of GlcNAc inner-membrane permeases. A neighbor-joining dendrogram was constructed using amino acid sequences of GlcNAc permeases. Branch labels indicate bootstrap value from 1,000 replicates. Protein accession numbers are given in brackets.

Our phenotypic data indicated at least two inner-membrane permeases, NagPsm and NagF, mediate GlcNAc influx in *S. maltophilia*, and neither appears to be a PTS-type transporter. Phylogenetic analysis placed NagPsm and NagF closer to NagP of *X. campestris* (4) than to the PTS-type transporter (*E. coli* NagE) (16) (Fig. 3). In *X. campestris*, GlcNAc imported by NagP is phosphorylated by cytoplasmic kinases NagK-IIA and NagK-IIB (4). By analogy, we hypothesized that *S. maltophilia* harbors a cytoplasmic GlcNAc kinase. The genome-wide search in strain *S. maltophilia* K279a identified Smlt4435 as a prime candidate: it encodes a 285-amino acid protein with 24% and 21% identity to NagK-IIA and NagK-IIB of *X. campestris*, respectively. We therefore designated Smlt4435 as NagK.

**Fig 4:**
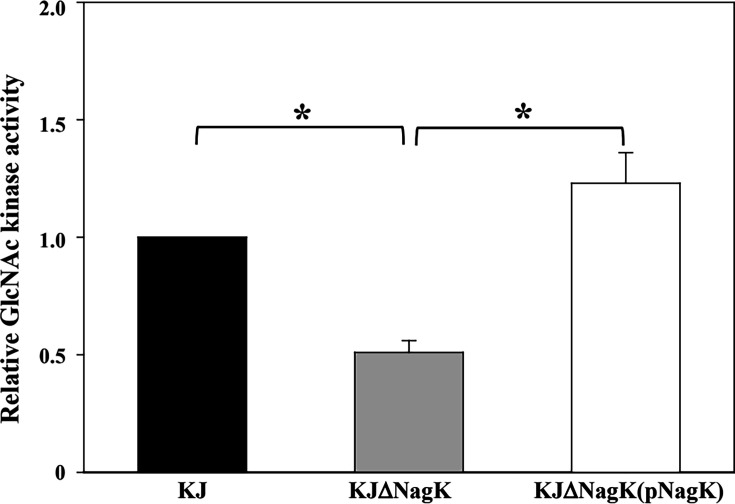
GlcNAc kinase activity. Cells were grown in lysogeny broth with 100 mM GlcNAc at 37°C for 15 h. Cell extracts were assayed for GlcNAc kinase activity by the coupled pyruvate kinase-lactate dehydrogenase method. Relative activity is expressed with KJ set to 1. Bars show means ± SEM from three independent experiments. *, *P* < 0.001(Student’s *t*-test).

An in-frame deletion of *nagK* was constructed and subjected to GlcNAc kinase activity assay, in which ATP was added to the reaction buffer as the phosphate donor. The GlcNAc kinase activity of KJΔNagK was approximately 50% of wild-type KJ, and activity was restored when *nagK* was supplied in *trans* on plasmid pNagK (Fig. 4). Deletion of *nagK* moderately impaired growth on XOLN-GlcNAc (Fig. 2), supporting a role for NagK in GlcNAc phosphorylation, while also indicating the presence of at least one additional kinase capable of phosphorylating GlcNAc.

### Amino sugar sensitivity is observed in *ΔnagB* and *ΔnagAΔnagA2* mutants, but not in *ΔnagA* or *ΔnagA2* mutants

**Fig 5:**
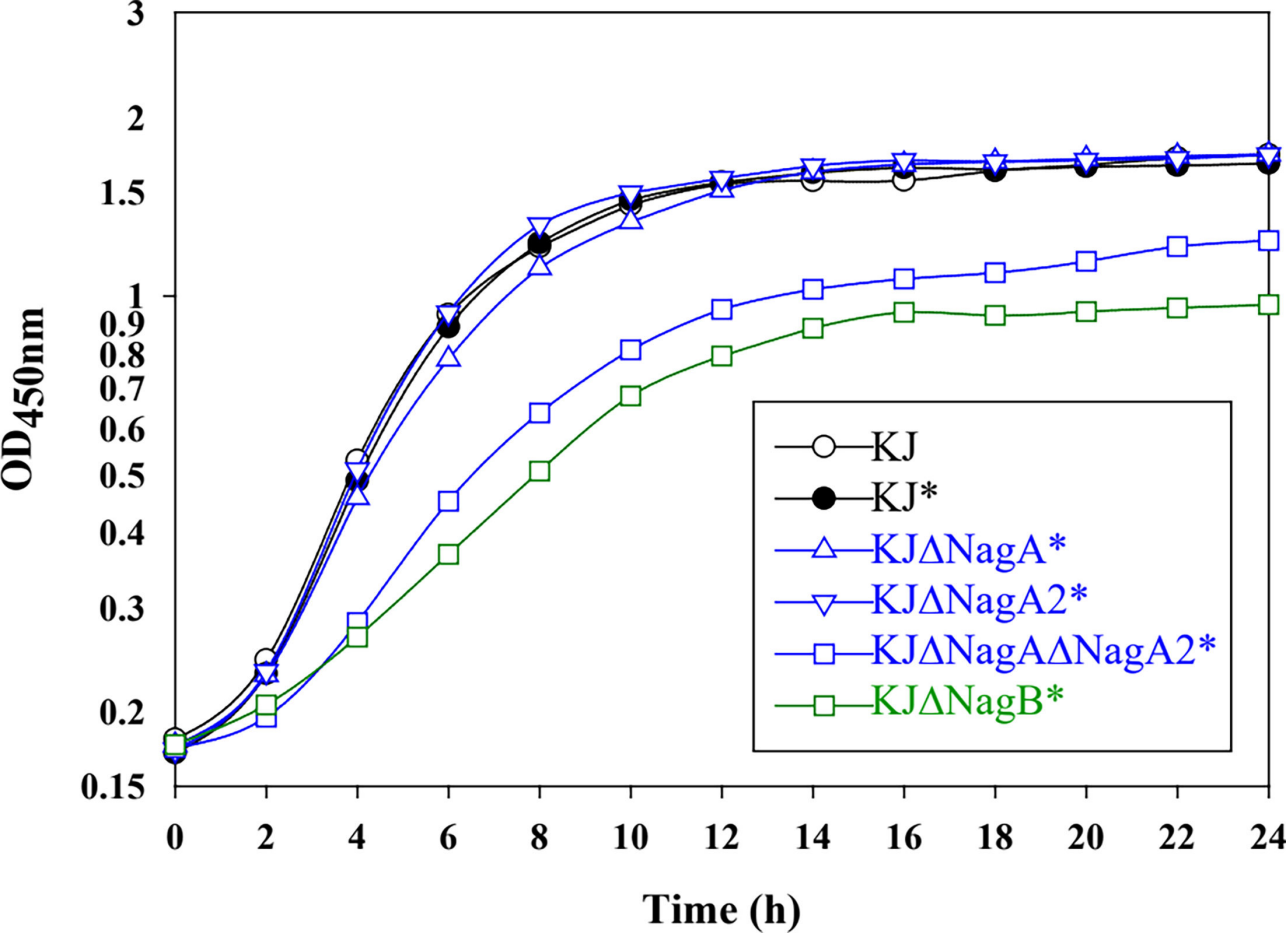
Amino sugar sensitivity in *S. maltophilia*. Overnight culture was inoculated into LB with or without 100 mM GlcNAc at an initial OD_450nm_ of 0.15, and growth was monitored by OD_450nm_. Graph is representative of at least three independent experiments. *, additive of 100 mM GlcNAc.

Amino sugar sensitivity describes growth inhibition in the presence of GlcNAc and was first described in *E. coli* (17). In *E. coli*, both *nagA* and *nagB* mutants show this phenotype, with the defect being less pronounced in the *nagB* mutant (17). To assess whether a similar phenomenon occurs in *S. maltophilia*, we compared the growth of wild-type KJ, KJΔNagA, KJΔNagA2, KJΔNagAΔNagA2, and KJΔNagB in lysogeny broth (LB) with or without 100 mM GlcNAc. All strains grew similarly in plain LB (representative data shown in Fig. 5). The addition of GlcNAc caused growth impairment in the *ΔnagAΔnagA2* and *ΔnagB* mutants (Fig. 5). Complementation of *ΔnagAΔnagA2* and *ΔnagB* mutants with plasmid pNagA, pNagA2, and pNagB, respectively, restored bacterial growth (Fig. S4). These evidences indicated that loss of GlcNAc-6P deacetylase and GlcN-6P deaminase leads to amino sugar sensitivity in *S. maltophilia*. Notably, unlike the pattern reported in *E. coli*, the amino sugar sensitivity was more pronounced in the *nagB* mutant in *S. maltophilia*.

### The *nag* operon is inducibly expressed by GlcNAc and GlcNAc-6P, which functions as the inducer

We previously demonstrated that the *nag* operon is negatively regulated by NagI, a LacI-family repressor (14). Because many carbohydrate regulators sense sugar derivatives via a C-terminal effector-binding domain (11), we tested whether GlcNAc itself or a derivative (e.g., GlcNAc-6P) serves as the NagI effector. Using the transcriptional reporter plasmid pNagP_xylE_ (a *nagP* promoter fusion to promoterless *xylE*) (14), we measured promoter activity in wild-type KJ and in the deletion mutants KJΔNagP, KJΔNagA, KJΔNagA2, KJΔNagF, and KJΔNagPΔNagF. Because KJΔNagB and KJΔNagAΔNagA2 display growth defects in GlcNAc-containing medium (Fig. 5), they were excluded from this assay; KJΔNagI(pNagP_xylE_) (14) was included as a derepressed control.

**Fig 6:**
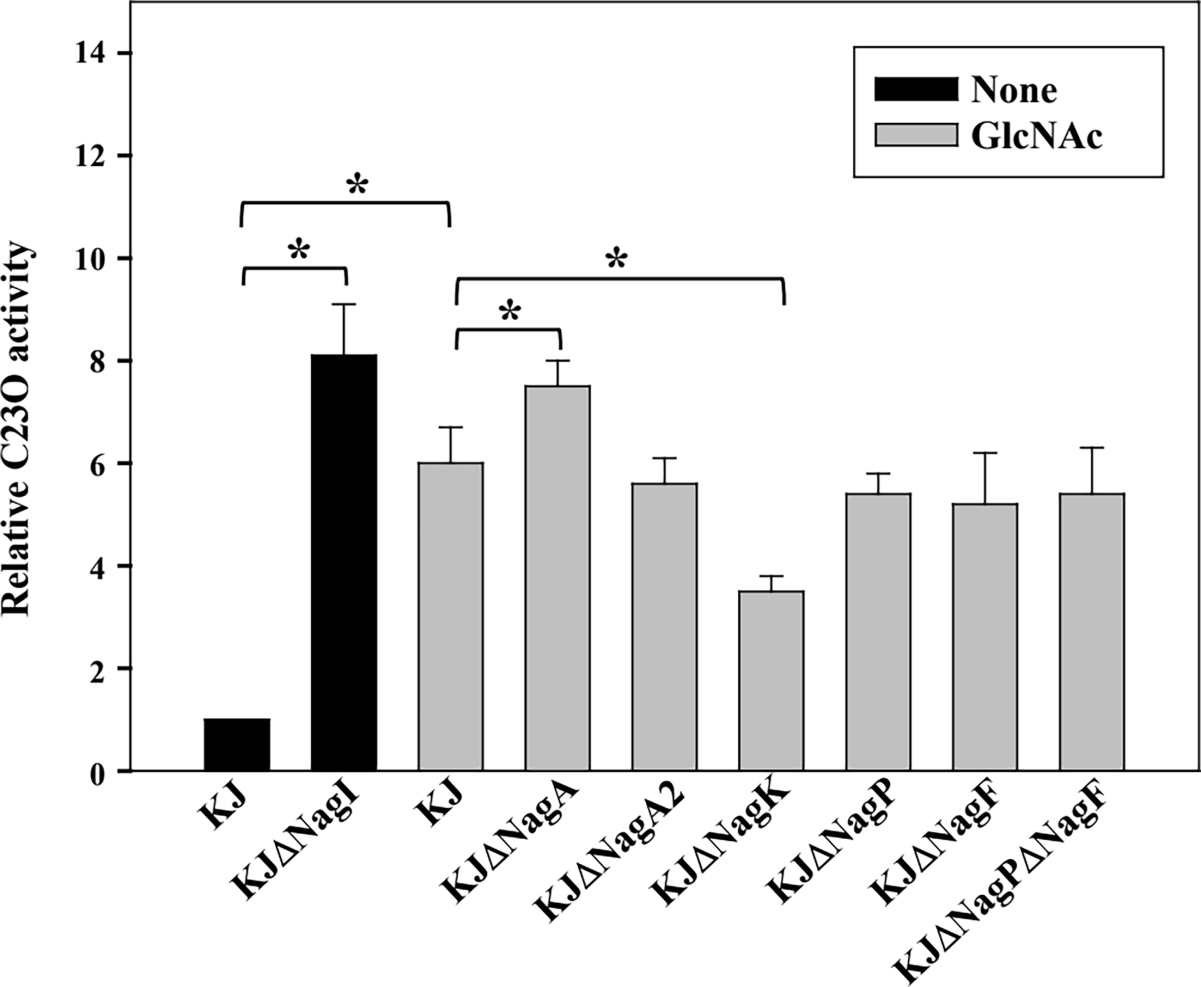
Regulation of *nag* operon expression. Plasmid pNagP_xylE_ was introduced into wild-type KJ and mutant strains. Cultures were grown in LB with or without GlcNAc for 5 h and catechol 2,3-dioxygenase (C23O) activities were measured. Bars are means ± SEM from three independent experiments. *, *P* < 0.001 (Student’s *t*-test).

As expected, *nag* expression increased in the *nagI* mutant (Fig. 6), confirming NagI’s role as a repressor (14). Upon GlcNAc challenge, promoter activity in most mutants resembled wild-type KJ, except that activity was elevated in KJΔNagA and reduced in KJΔNagK (Fig. 6). These changes are consistent with GlcNAc-6P being the inducing ligand sensed by NagI.

### Effect of *nag* operon upregulation on colistin and β-lactam bacterial susceptibility to colistin and β-lactam, MD tolerance, and swimming

**Fig 7:**
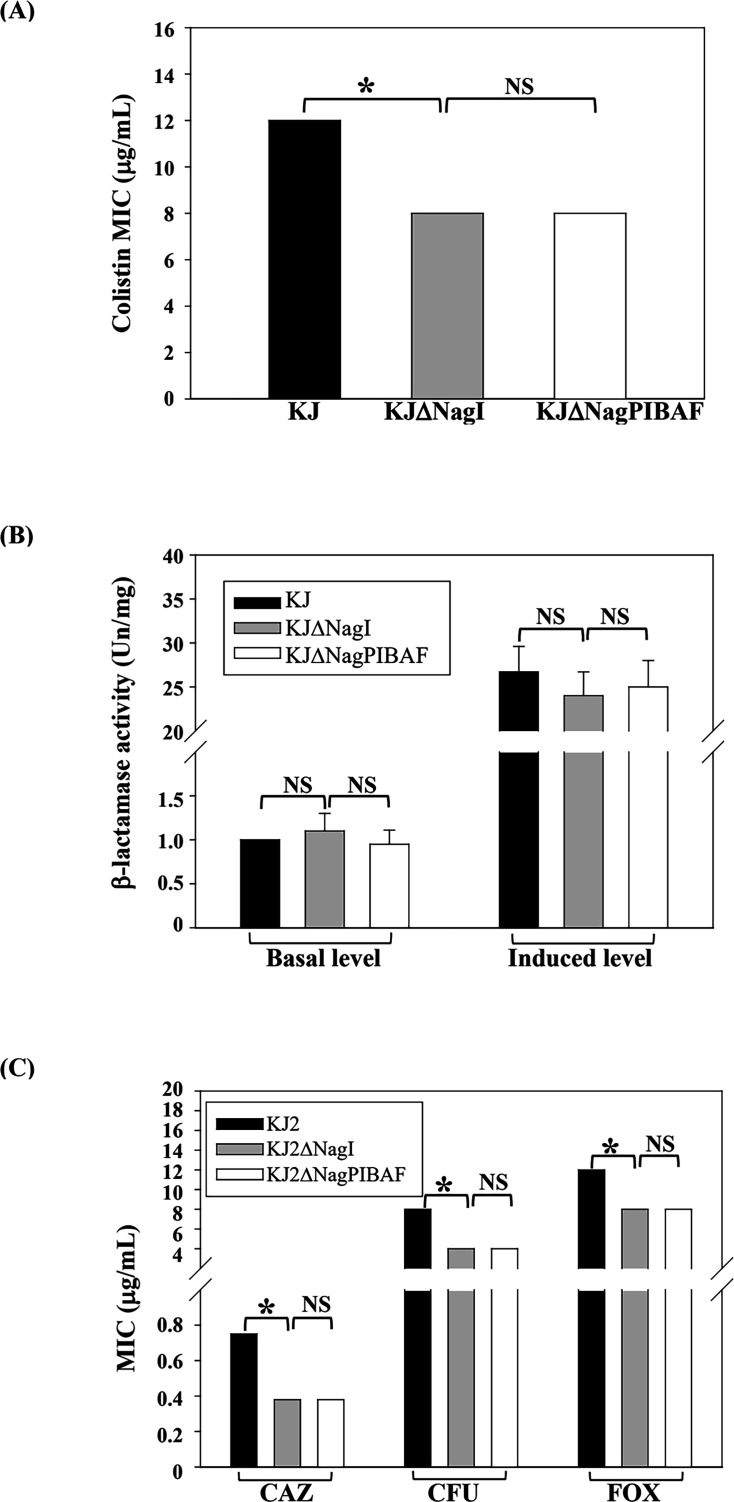
Role of *nagPIBAF* upregulation in susceptibility to colistin and β-lactams. Data are means of three independent experiments; error bars indicate standard deviations. *, *P* ≤ 0.05, (Student’s *t* test); NS, non-significant. (**A**) Colistin MICs of wild-type KJ and its derived mutants, determined by *E*-test. (**B**) Basal and induced β-lactamase activities in whole-cell extracts measured by nitrocefin hydrolysis; cells were treated with or without 50 μg/mL CAZ for 30 min. (**C**) β-lactam susceptibility of β-lactamase-null parental strain KJ2 and its derived mutants, determined by *E*-test. CAZ, ceftazidime; CFU, cefuroxime; FOX, cefoxitin.

Because colistin and β-lactams target LPS and PG synthesis, respectively, and because *nagPIBAF* operon links GlcNAc metabolism to these biosynthetic pathways (Fig. 1A), we evaluated whether *nag* operon upregulation affects susceptibility to these antibiotics. We used KJΔNagI (representing *nag* upregulation) and KJΔNagPIBAF (representing loss of *nag* expression) for these assays. KJΔNagI showed a lower colistin MIC than wild-type KJ; nevertheless, colistin MICs for KJΔNagI and KJΔNagPIBAF were comparable (Fig. 7A).

Because β-lactamase expression is the primary determinant of β-lactam resistance in *S. maltophilia* (18), we first compared β-lactamase activities. Wild-type KJ, KJΔNagI, and KJΔNagPIBAF exhibited similar basal and induced β-lactamase activities (Fig. 7B), indicating that *nag* operon upregulation does not alter β-lactamase expression. To assess non-β-lactamase-mediated β-lactam resistance, we employed the β-lactamase-null mutant parental strain KJ2 (19). Compared with KJ2, KJ2ΔNagI was more susceptible to β-lactams, but KJ2ΔNagI and KJ2ΔNagPIBAF showed comparable susceptibilities (Fig. 7C). Together, these data indicate that *nag* operon upregulation *per se* has little effect on susceptibility to colistin and β-lactams, although other NagI-regulated genes outside the *nagPIBAF* circuit may influence susceptibility to colistin and β-lactam.

Because *nagPIBAF* expression channels metabolism toward glycolysis (Fig. 1), we hypothesized that its overexpression might elevate tricarboxylic acid cycle and electron transport chain activity (Fig. 1), potentially increasing reactive oxygen species and ATP production. Generally, bacterial motility is driven by energy from ATP (20). To test whether such metabolic reprogramming affects oxidative stress tolerance or swimming motility, we measured menadione (MD) IC_50_ and swimming motility. MD is commonly used as a superoxide generator in the study of oxidative stress responses. The MD IC_50_ values and swimming diameters were comparable between KJΔNagI and KJΔNagPIBAF (Fig. S5), indicating that *nagPIBAF* upregulation does not substantially alter MD tolerance and swimming under our assay conditions.

### Impact of GlcNAc utilization on antibiotic susceptibility, MD tolerance, and swimming motility

Because GlcNAc is abundant in infection niches (21), we tested how GlcNAc utilization affects antibiotic susceptibility, oxidative stress tolerance, and swimming motility in clinical isolates. Besides KJ, six randomly chosen ceftazidime (CAZ)-susceptible *S. maltophilia* clinical isolates were assayed. In the presence of GlcNAc, 71.4% (5/7) and 57.1% (4/7) of isolates displayed increased MICs to colistin and CAZ, respectively (Table 1). To clarify that the increase in MIC was due to GlcNAc metabolism rather than a general osmotic effect, we used SEM to observe the morphology of isolates YT-4, YT-17, and YT-119 in the presence and absence of GlcNAc, since the MICs of these isolates against colistin and CAZ were increased in the presence of GlcNAc (Table 1). The results showed that GlcNAc did not affect the appearance of the bacterial cells (Fig. S6), ruling out the consideration of osmotic effect. All isolates had similar MD IC_50_ values regardless of GlcNAc (Table 1), indicating little effect on oxidative stress tolerance. By contrast, most isolates showed enhanced swimming in the presence of GlcNAc, with pronounced increases observed in KJ, YT-17, YT-84, and YT-119 (Table 1). To further clarify that these phenotype alterations result from the metabolic effect of GlcNAc, rather than from the signaling of GlcNAc, we selected the mutants with compromised ability in GlcNAc metabolism and without “amino sugar sensitivity” as controls to decouple signaling from metabolic effect. Based on the results of Fig. 2 and 5, KJΔNagPΔNagF and KJΔNagA were selected as controls for the assessment in swimming motility. KJ cells increased swimming motility in the presence of GlcNAc; however, this phenotype was not obvious in KJΔNagPΔNagF and KJΔNagA cells (Table S3), supporting that GlcNAc metabolism, rather than GlcNAc signaling, contributes to enhance swimming motility.

**TABLE 1:** Impact of GlcNAc on bacterial susceptibility to CAZ and colistin, MD tolerance, and swimming motility

Strain	Colistin MIC (μg/mL)		CAZ MIC (μg/mL)		MD IC_50_		Swimming zone (mm)	
	GlcNAc(−)	GlcNAc(+)	GlcNAc(−)	GlcNAc(+)	GlcNAc(−)	GlcNAc(+)	GlcNAc(−)	GlcNAc(+)
KJ	8	12	>256	>256	34 ± 4	35 ± 4	34 ± 2	40 ± 2
KJ2	ND^*a*^	ND^*a*^	0.38	0.38	ND^*a*^	ND^*a*^	ND^*a*^	ND^*a*^
YT-4	3	6	0.75	1.0	27 ± 3	26 ± 3	34 ± 2	36 ± 2
YT-8	12	12	1.0	1.5	28 ± 2	30 ± 4	24 ± 1	28 ± 1
YT-12	2	2	1.0	1.0	23 ± 3	25 ± 3	47 ± 2	49 ± 2
YT-17	4	6	6	12	23 ± 3	21 ± 3	21 ± 2	30 ± 1
YT-84	12	16	1.5	1.5	37 ± 3	37 ± 4	42 ± 2	47 ± 2
YT-119	6	16	1	1.5	28 ± 3	27 ± 3	38 ± 2	46 ± 2

^*a*^ ND, non-determined.

## DISCUSSION

The GlcNAc utilization pathway generally comprises transporters, kinases, a GlcNAc-6P deacetylase (NagA), and a GlcN-6P deaminase (NagB). While transport and phosphorylation components differ among species, NagA and NagB are broadly conserved (4, 8, 9). Consistent with this, *S. maltophilia* strains lacking NagB cannot use GlcNAc as the sole carbon source (Fig. 2). Unlike many organisms, however, *S. maltophilia* carries two *nagA* homologs—*nagA* and *nagA2*—whose products are functionally redundant for GlcNAc deacetylation.

We identified two inner-membrane permeases, NagPsm and NagF, responsible for GlcNAc utilization in *S. maltophilia*, both of which are non-PTS transporters. Phylogenetic analysis groups NagPsm and NagF with *X. campestris* NagP rather than with *E. coli* NagE (PTS transporter; Fig. 3). We also identified NagK as a cytoplasmic GlcNAc kinase, although residual kinase activity in the Δ*nagK* mutant suggests the presence of an additional kinase. Notably, NagF appears to constitute a previously uncharacterized family of GlcNAc transporters: it shares 51% similarity and 35% identity with MurT from *Tannerella forsythia*, a MurNAc permease (15), suggesting that NagF may accept substrates beyond GlcNAc. We also observed that the growth defect was more severe in KJΔNagP than in KJΔNagF (Fig. 2), suggesting that NagPsm makes the larger contribution to GlcNAc transport. Therefore, NagPsm seems to be the primary permease and NagF as a likely auxiliary one. The fact that the *nagPsm*/*nagF* double mutant retained some ability to grow on XOLN-GlcNAc (Fig. 2) implies at least one additional GlcNAc permease remains to be identified in *S. maltophilia*.

Regulatory strategies for GlcNAc utilization vary across bacteria. In *E. coli*, *nagE-nagBACD* expression is controlled in parallel by the ROK family regulator NagC (22, 23) (Fig. 1B). GntR family regulators deploy in several bacteria, e.g., NagQ in *Xanthomonas campestris* (4) (Fig. 1B), NagR in *Caulobacter crescentus* (9) and *Streptococcus mutans* (24), and DasR in *Streptomyces coelicolor* (25). In *S. maltophilia*, we show that a LacI-family regulator, NagI, represses *nag* operon and that GlcNAc-6P serves as the inducing ligand, a regulatory logic shared with multiple other species (4, 9, 25, 26) (Fig. 6). The evolutionary relationship among these regulators was present in Fig. S7.

Although *nagPIBAF* upregulation had limited impact on colistin and β-lactam susceptibility, MD tolerance, and swimming under our assay conditions (Fig. 7 and Fig. S1), NagI deletion altered colistin and β-lactam susceptibilities in ways that were independent of the *nagPBAF* circuit (Fig. 7). Given the growing recognition that metabolism can influence antibiotic resistance (27, 28) and that LacI regulators broadly modulate carbohydrate metabolism (11), it is plausible that deletion of *nagI* triggers wider metabolic reprogramming beyond the *nagPBAF* circuit, thereby affecting antibiotic susceptibility via indirect routes.

GlcNAc utilization can increase the intracellular UDP-GlcNAc level (Fig. 1). UDP-GlcNAc can serve as the foundation for the synthesis of PG and LPS Lipid A. For PG synthesis, UDP-GlcNAc is subsequently processed to UDP-GlcNAc-pentapeptide, Lipid I, and Lipid II, which is then flipped to the periplasm for incorporation into the pre-exist PG (29). UDP-GlcNAc-pentapeptide acts as a corepressor for the AmpR transcription regulator to repress the expression of L1 and L2 β-lactamases (30). On the other hand, GlcNAc is a critical precursor for lipid A biosynthesis. Lipid A modification by 4-amino-4-deoxy-L-arabinose is the major mechanism responsible for colistin resistance in gram-negative bacteria (31). This can be the reason why GlcNAc utilization is linked to β-lactam and colistin resistance in some *S. maltophilia* isolates (Table 1).

GlcNAc can also act as a signaling molecule modulating diverse bacterial behaviors, including morphogenesis, virulence, pilus expression, flagellar motility, sporulation, and secondary metabolite production (32–35), and it can affect microbial community composition (36, 37). In our clinical isolates, the presence of GlcNAc tended to increase resistance to CAZ and colistin and to enhanced swimming motility, although responses varied among isolates (Table 1). Enhanced swimming motility may contribute to virulence (38). The underlying mechanisms responsible for these phenotype changes are not immediately clear in this study; a whole genome-wide comparative analysis (such as RNAseq transcriptome and proteomics) between bacteria with and without GlcNAc treatment can be a strategy to explore in the future. Our findings in this study revealed that GlcNAc can enhance antibiotic resistance and virulence in some clinical *S. maltophilia* isolates. GlcNAc is widely used as a dietary supplement for joint health (39, 40); these findings suggest that careful consideration is warranted regarding potential unintended effects of GlcNAc on pathogens and microbiota.

## MATERIALS AND METHODS

### Bacterial strains, plasmids, and medium

Strains and plasmids used in this study are listed in Table S1. XOLN medium contained 0.0625% yeast extract, 0.0625% tryptone, 0.07% K_2_HPO_4_, 0.02% KH_2_PO_4_, 0.1% (NH_4_)_2_SO_4_, 0.01% MgCl_2_⋅6H_2_O, 0.001% FeSO_4_⋅7H_2_O, and 0.0001% MnCl_2_, at pH 7.15.

### In-frame deletion mutant construction

An allelic replacement strategy was used to generate in-frame deletion mutants. For constructing pΔNagB and pΔNagA, intact *nagB* and *nagA* were PCR-amplified using primer pairs NagB-F/R and NagA-F/R (Table S2) and cloned into pEX18Tc (41) to yield pNagB and pNagA. PstI-PstI fragments of 201 bp and 426 bp were removed from pNagB and pNagA to generate pΔNagB and pΔNagA (Table S1). Two flanking fragments bracketing the intended deletion regions were amplified and cloned into pEX18Tc to create pΔNagP, pΔNagF, pΔNagA2, and pΔNagK using primer pairs NagPN-F/R and NagPC-F/R, NagFN-F/R and NagFC-F/R, NagA2N-F/R and NagA2C-F/R, and NagKN-F/R and NagKC-F/R, respectively (Table S2). These pEX18Tc-derived plasmids were mobilized into *S. maltophilia* by conjugation. Transconjugants selection and mutant confirmation followed previously described procedures (42). Double mutants were constructed sequentially from single mutants using the same protocol.

### Growth curves

Overnight cultures were inoculated into fresh medium at an initial OD_450nm_ of 0.15. Bacterial growth was monitored by measuring OD_450nm_ at fixed time intervals.

### GlcNAc kinase activity assay

GlcNAc kinase activity was measured using a coupled pyruvate kinase-lactate dehydrogenase assay with minor modifications (4). Overnight cultures were inoculated to 50 mL LB containing 100 mM GlcNAc and incubated at 37°C for 15 h. Cells were pelleted at 4,500 rpm for 15 min, resuspended in 8 mL resuspension buffer (0.05 mM Tris-HCl, pH 8.0; 13.3 mM MgCl_2_; 0.1 mM EDTA; 1 mM dithiothreitol), and disrupted by French press. After centrifugation at 13,000 rpm for 10 min, supernatants were pre-warmed at 37°C for 5 min. Fifty microliter of supernatant was added to 450 μL reaction buffer (100 mM Tris HCl, pH 7.5; 10 mM MgCl_2_; 1 mM phosphoenolpyruvate; 4 mM ATP; 0.2 mM NADH; 10 mM GlcNAc; 4 U lactate dehydrogenase; 4 U pyruvate kinase), and the decrease in absorbance at 340 nm was monitored. Protein concentration was measured by Bio-Rad protein assay. One unit (U) was defined as the amount of enzyme that converts 1 nmol NADH into NAD^+^ per minute. Specific activity (U/mg) was calculated as units per milligram of proteins. Data represent three independent experiments.

### Catechol 2,3-dioxygenase activity assay

Catechol 2,3-dioxygenase activity (encoded by *xylE*) was assayed using catechol as substrate as previously described (18). One unit (Uc) was defined as the amount of enzyme converting 1 nmol catechol per minute. Specific activity (Uc/OD_450nm_) was expressed as units per OD_450nm_ of cells. All assays were performed in triplicate.

### β-lactamase activity assay

Overnight cultures were diluted into fresh LB to an initial OD_450nm_ of 0.15. After 3 h of culture at 37°C, 50 μg/mL CAZ was added for 30 min to induce β-lactamase. Basal and induced β-lactamase activities were determined using nitrocefin as substrate (42).

### Antibiotic susceptibility by *E*-test

Susceptibility to CAZ and colistin was determined using *E*-test strip (Liofilchem, Italy) following the manufacturer’s instructions. MICs were read at the intersection of the inhibition ellipse with the strip.

### Determination of MD IC_50_

MD IC_50_ (the MD concentration causing 50% loss of viability) was determined by plating serial dilutions of 10^6^ CFU/μL onto LB agar containing increasing concentrations of MD (0–60 μg/mL) with or without GlcNAc. After 24 h incubation at 37°C, CFUs were counted, and relative viability was calculated against the MD-free control (100%). MD IC_50_ values were derived by curve fitting of viability versus MD concentration.

### Swimming motility

One microliter of logarithmic-phase culture was spotted onto swimming agar (1% tryptone, 0.5% NaCl, and 0.15% agar) with or without GlcNAc. Plates were incubated at 37°C for 48 h, and swimming zone diameters (mm) were recorded. Experiments were performed in triplicate.

## SUPPLEMENTAL MATERIAL

Fig. S1 spectrum.03167-25-s0001.pdf Polar effect evaluation of *nagPIBAF* operon-associated mutants.

Fig. S2 spectrum.03167-25-s0002.pdf Complementation assay of *nagK*, *nagP*, *nagB*, *nagA*, and *nagF* mutants utilizing GlcNAc as the sole carbon source for growth.

Fig. S3 spectrum.03167-25-s0003.pdf Complementation assay of *nagA* and *nagA2* mutant utilizing GlcNAc as the sole carbon source for growth.

Fig. S4 spectrum.03167-25-s0004.pdf Complementation assay for “amino sugar sensitivity” phenomenon in *S. maltophilia*.

Fig. S5 spectrum.03167-25-s0005.pdf Role of *nagPIBAF* upregulation in MD tolerance and swimming motility.

Fig. S6 spectrum.03167-25-s0006.pdf Morphology of isolates YT-4, YT-17, and YT-119 grown in the presence and absence of GlcNAc.

Fig. S7 spectrum.03167-25-s0007.pdf Phylogenetic analysis of the transcriptional regulators of GlcNAc utilization.

Table S1 spectrum.03167-25-s0008.pdf Bacterial strains and plasmids used in this study.

Table S2 spectrum.03167-25-s0009.pdf Primers used in this study.

Table S3 spectrum.03167-25-s0010.pdf Impact of GlcNAc on swimming motility of KJ, KJΔNagA, and KJΔNagPΔNagF.

ASM does not own the copyrights to Supplemental Material that may be linked to, or accessed through, an article. The authors have granted ASM a non-exclusive, world-wide license to publish the Supplemental Material files. Please contact the corresponding author directly for reuse.
