# Supplementary material for: N-acetylglucosamine utilization and impact on antibiotic susceptibility, oxidative stress tolerance, and swimming in Stenotrophomonas maltophilia
Source: Microbiol Spectr. 2026 Mar 16;14(4):e03167-25. doi: 10.1128/spectrum.03167-25 (PMC13055268; doi:10.1128/spectrum.03167-25)
Supplement: Fig. S1 — Polar effect evaluation of nagPIBAF operon-associated mutants. [file spectrum.03167-25-s0001.pdf]

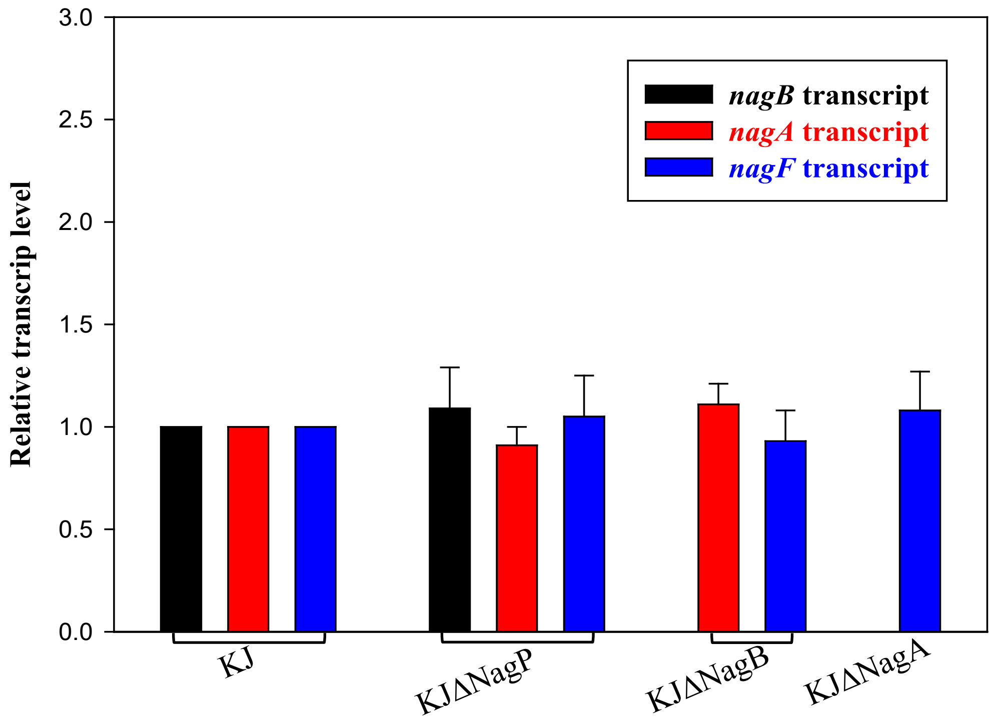

Fig. S1. Polar effect evaluation of *nagPIBAF* operon-associated mutants. Logarithmically grown cells were harvested. The transcript levels of *nagB*, *nagA*, and *nagF* were determined by qRT-PCR and normalized to 16S rRNA. Relative transcript level was calculated using the transcript level of KJ as 1 Data are mean ± SD of three independent experiments; significance was determined using a two-tailed Student's *t*-test (*$P \leq 0.05$).
