# Supplementary material for: N-acetylglucosamine utilization and impact on antibiotic susceptibility, oxidative stress tolerance, and swimming in Stenotrophomonas maltophilia
Source: Microbiol Spectr. 2026 Mar 16;14(4):e03167-25. doi: 10.1128/spectrum.03167-25 (PMC13055268; doi:10.1128/spectrum.03167-25)
Supplement: Fig. S3 — Complementation assay of nagA and nagA2 mutant utilizing GlcNAc as the sole carbon source for growth. [file spectrum.03167-25-s0003.pdf]

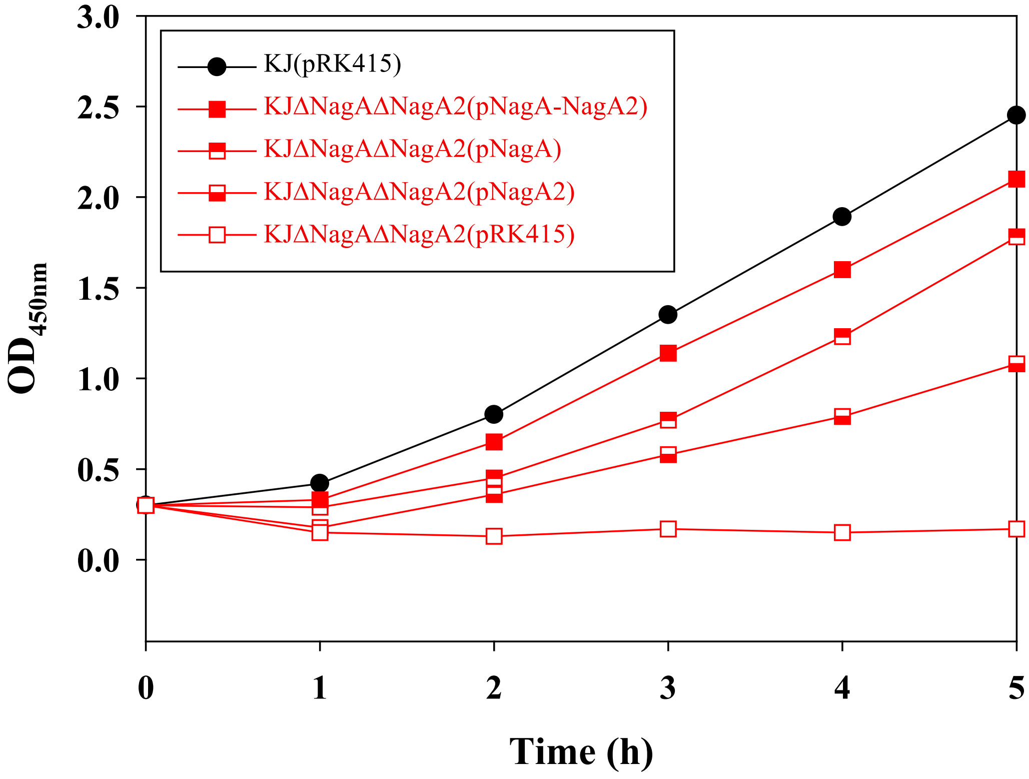

Fig. S3. Complementation assay of *nagA* and *nagA2* mutant utilizing GlcNAc as the sole carbon source for growth. Overnight cultures were inoculated into XOLN medium with 100 mM GlcNAc at an initial OD_{450nm} of 0.15; growth was followed by OD_{450nm} readings. Graph is representative of at least three independent experiments.
