# Supplementary material for: N-acetylglucosamine utilization and impact on antibiotic susceptibility, oxidative stress tolerance, and swimming in Stenotrophomonas maltophilia
Source: Microbiol Spectr. 2026 Mar 16;14(4):e03167-25. doi: 10.1128/spectrum.03167-25 (PMC13055268; doi:10.1128/spectrum.03167-25)
Supplement: Fig. S4 — Complementation assay for “amino sugar sensitivity” phenomenon in S. maltophilia. [file spectrum.03167-25-s0004.pdf]

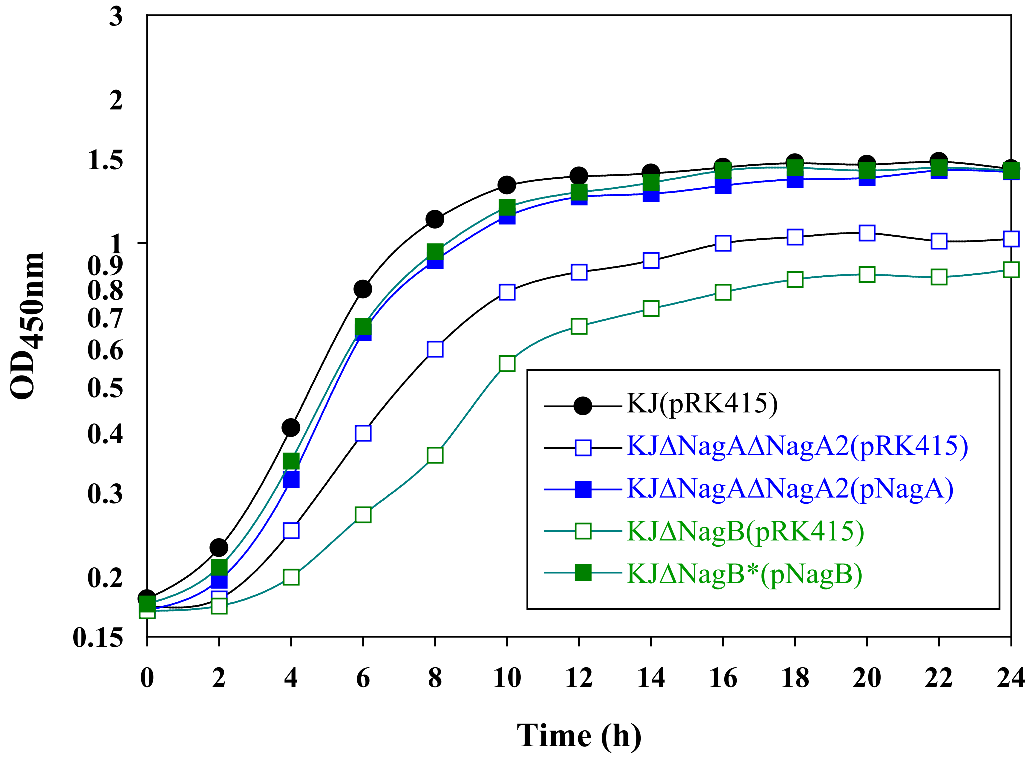

Fig. S4. Complementation assay for "amino sugar sensitivity" phenomenon in *S. maltophilia*. The overnight culture of bacteria tested was inoculated into fresh LB medium with 100 mM GlcNAc at an initial OD_{450nm} of 0.15. The bacterial growth was monitored by recording the OD_{450nm}. The graph is representative of at least three replicated experiments.
