# Supplementary material for: N-acetylglucosamine utilization and impact on antibiotic susceptibility, oxidative stress tolerance, and swimming in Stenotrophomonas maltophilia
Source: Microbiol Spectr. 2026 Mar 16;14(4):e03167-25. doi: 10.1128/spectrum.03167-25 (PMC13055268; doi:10.1128/spectrum.03167-25)
Supplement: Fig. S5 — Role of nagPIBAF upregulation in MD tolerance and swimming motility. [file spectrum.03167-25-s0005.pdf]

(A)

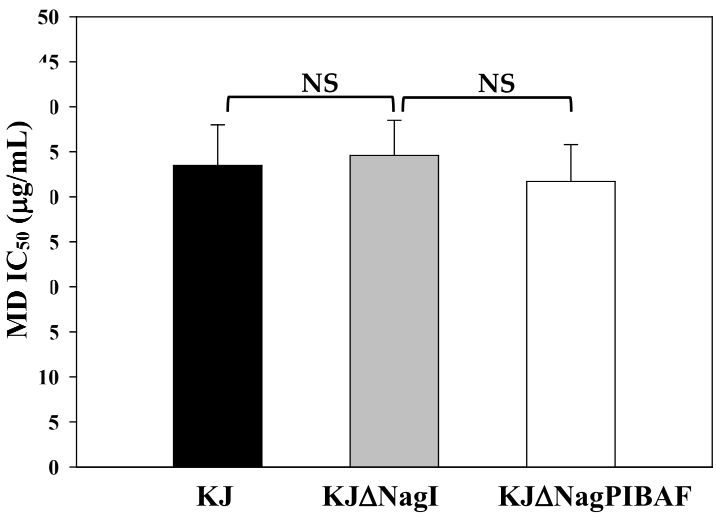

(B)

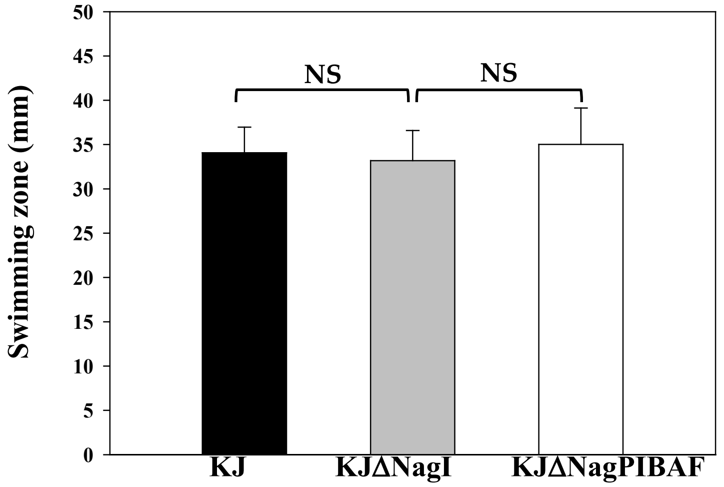

Fig. S5. Role of *nagPIBAF* upregulation in MD tolerance and swimming motility. Data is the means from three independent experiments. The error bars represent the standard deviations for triplicate samples. *, $P \leq 0.05$, significance calculated by Student's *t* test. NS, non-significant. (A) Role of *nagPIBAF* upregulation in MD tolerance. (B) Role of *nagPIBAF* upregulation in swimming motility.
