# Supplementary material for: N-acetylglucosamine utilization and impact on antibiotic susceptibility, oxidative stress tolerance, and swimming in Stenotrophomonas maltophilia
Source: Microbiol Spectr. 2026 Mar 16;14(4):e03167-25. doi: 10.1128/spectrum.03167-25 (PMC13055268; doi:10.1128/spectrum.03167-25)
Supplement: Fig. S6 — Morphology of isolates YT-4, YT-17, and YT-119 grown in the presence and absence of GlcNAc. [file spectrum.03167-25-s0006.pdf]

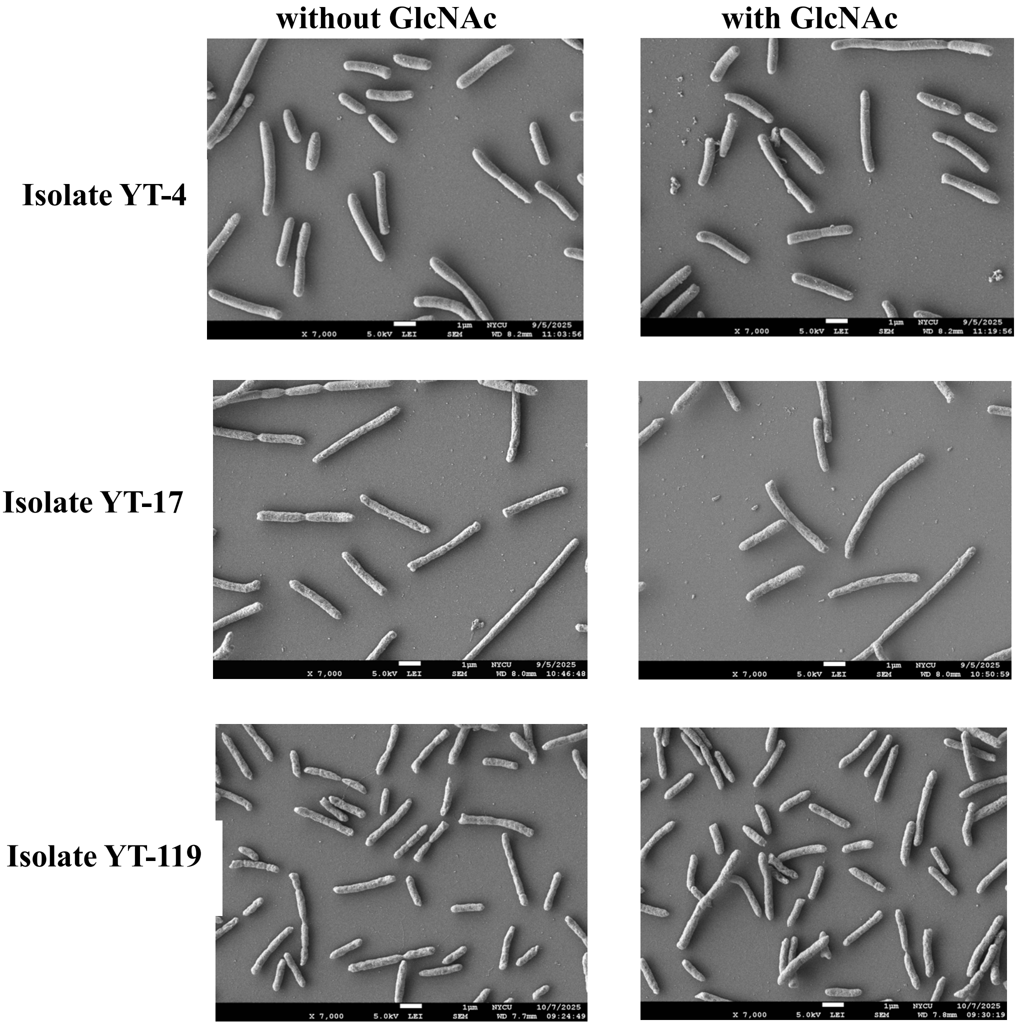

Fig. S6. Morphology of isolates YT-4, YT-17, and YT-119 grown in the presence and absence of GlcNAc. Overnight-cultured bacterial cells were inoculated into fresh LB broth with or without 100 mM GlcNAc. After 5-h incubation, exponentially grown bacterial cells were harvested and observed by scanning electron microscopy.
