# Supplementary material for: N-acetylglucosamine utilization and impact on antibiotic susceptibility, oxidative stress tolerance, and swimming in Stenotrophomonas maltophilia
Source: Microbiol Spectr. 2026 Mar 16;14(4):e03167-25. doi: 10.1128/spectrum.03167-25 (PMC13055268; doi:10.1128/spectrum.03167-25)
Supplement: Fig. S7 — Phylogenetic analysis of the transcriptional regulators of GlcNAc utilization. [file spectrum.03167-25-s0007.pdf]

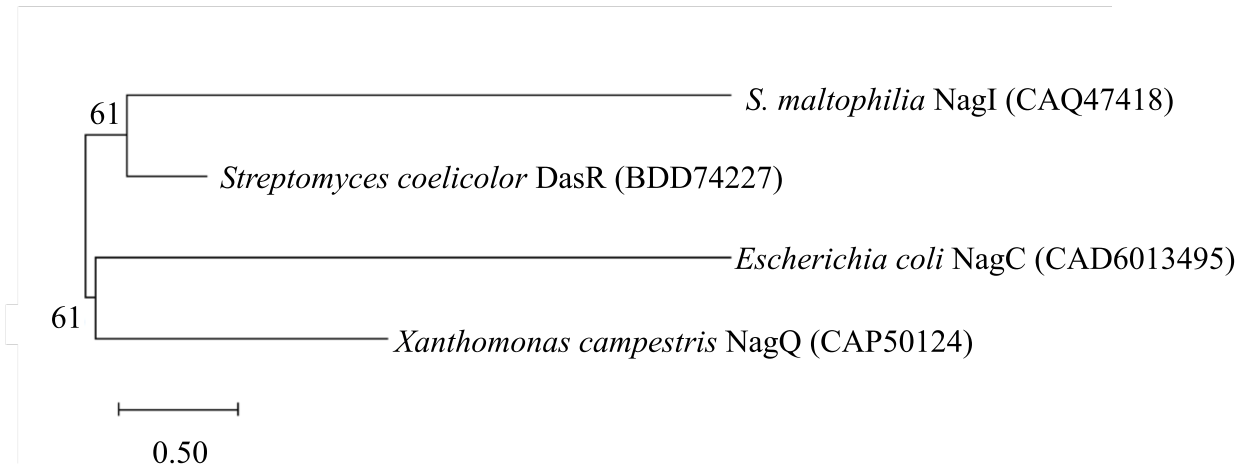

Fig. S7. Phylogenic analysis of the transcriptional regulators of GlcNAc utilization. A neighbor-joining dendrogram was constructed using amino-acid sequences the transcriptional regulators of GlcNAc utilization. Branches labels indicate bootstrap value from 1,000 replicates. Protein accession numbers are given in brackets.
