# Supplementary material for: N-acetylglucosamine utilization and impact on antibiotic susceptibility, oxidative stress tolerance, and swimming in Stenotrophomonas maltophilia
Source: Microbiol Spectr. 2026 Mar 16;14(4):e03167-25. doi: 10.1128/spectrum.03167-25 (PMC13055268; doi:10.1128/spectrum.03167-25)
Supplement: Table S1 — Bacterial strains and plasmids used in this study. [file spectrum.03167-25-s0008.pdf]

Table S1 Bacterial strains and plasmids used in this study

Strain or plasmid	Genotype or properties	Reference
S. maltophilia		
KJ	A *S. maltophilia* clinical isolate	(1)
KJ2	*S. maltophilia* KJ mutant of *L1* and *L2* genes; *ΔL1, ΔL2*	(2)
YT-4	A *S. maltophilia* clinical isolate	This study
YT-8	A *S. maltophilia* clinical isolate	This study
YT-12	A *S. maltophilia* clinical isolate	This study
YT-17	A *S. maltophilia* clinical isolate	This study
YT-84	A *S. maltophilia* clinical isolate	This study
YT-119	A *S. maltophilia* clinical isolate	This study
KJΔNagP	*S. maltophilia* KJ mutant of *nagP* gene; *ΔnagP*	This study
KJΔNagI	*S. maltophilia* KJ mutant of *nagI* gene; *ΔnagI*	(3)
KJΔNagB	*S. maltophilia* KJ mutant of *nagB* gene; *ΔnagB*	This study
KJΔNagA	*S. maltophilia* KJ mutant of *nagA* gene; *ΔnagA*	This study
KJΔNagF	*S. maltophilia* KJ mutant of *nagF* gene; *ΔnagF*	This study
KJΔNagPΔNagF	*S. maltophilia* KJ mutant of *nagP* and *nagF* genes; *ΔnagP, ΔnagF*	This study
KJΔNagPIBAF	*S. maltophilia* KJ mutant of *nagPIBAF* operon; *ΔnagPIBAF*	(3)
KJΔNagA2	*S. maltophilia* KJ mutant of *nagA2* gene; *ΔnagA2*	This study
KJΔNagAΔNagA2	*S. maltophilia* KJ mutant of *nagA* and *nagA2* genes; *ΔnagA, ΔnagA2*	This study
KJΔNagK	*S. maltophilia* KJ mutant of *nagK* gene; *ΔnagK*	This study
E. coli		
DH5α	F- φ80d*lacZΔM15* Δ(*lacZYA-argF*)*U169 deoR recA1 endA1 hsdR17* ($r_k^- m_k^+$) *phoA supE44λ thi-1 gyrA96 relA1*	Invitrogen
S17-1	λpir^+ mating strain	(4)
**Plasmids**		
pEX18Tc	*sacB oriT*, Tc^r	(5)
pRK415	Mobilizable broad-host-range plasmid cloning vector, RK2 origin; Tc^r	(6)
pΔNagP	pEX18Tc with an internal deleted *nagP* gene; Tc^r	This study
pΔNagB	pEX18Tc with an internal deleted *nagB* gene;	This study

	Tc^r	
pΔNagA	pEX18Tc with an internal deleted *nagA* gene; Tc^r	This study
pΔNagF	pEX18Tc with an internal deleted *nagF* gene; Tc^r	This study
pΔNagA2	pEX18Tc with an internal deleted *nagA2* gene; Tc^r	This study
pΔNagK	pEX18Tc with an internal deleted *nagK* gene; Tc^r	This study
$pNagP_{xylE}$	pRK415 with a 300-bp DNA fragment upstream from the *nagP* start codon and a P_{nagP}::*xylE* transcriptional fusion	(3)

1. Hu RM, Huang KJ, Wu LT, Hsiao YJ, Yang TC. 2008. Induction of L1 and L2 β-lactamases of *Stenotrophomonas maltophilia*. *Antimicrob Agents Chemother* 52:1198-1200.
2. Chen CH, Huang CC, Chung TC, Hu RM, Huang YW, Yang TC. 2011. Contribution of resistance-nodulation-division efflux pump operon *smeU1-V-W-U2-X* to multidrug resistance of *Stenotrophomonas maltophilia*. *Antimicrob Agents Chemother* 55:5826-5833.
3. Yang TC, Wu SC, Yeh TY, Lu HF, Lin YT, Li LH. 2025. *NagPIBAF* upregulation and *ompO* downregulation compromise oxidative stress tolerance of *Stenotrophomonas maltophilia. BMC Microbiol.* 25:122.
4. Simon R, O'Connell M, Labes M, Puhler A. 1986. Plasmid vector for the genetic analysis and manipulation of *Rhizobia* and other Gram-negative bacteria. *Methods Enzymol* 118:640-659.
5. Hoang TT, Karkhoff-Schweizer RR, Kutchma AJ, Schweizer HP. 1998. A broad-host-range Flp-FRT recombination system for site-specific excision of chromosomally-located DNA sequences: application for isolation of unmarked *Pseudomonas aeruginosa* mutants. *Gene* 212:77-86.
6. Keen NT, Tamaki S, Kobayashi D, Trollinger D. 1998. Improved broad-host-range plasmids for DNA cloning in gram-negative bacteria. *Gene* 70:191-197.
