# Supplementary material for: N-acetylglucosamine utilization and impact on antibiotic susceptibility, oxidative stress tolerance, and swimming in Stenotrophomonas maltophilia
Source: Microbiol Spectr. 2026 Mar 16;14(4):e03167-25. doi: 10.1128/spectrum.03167-25 (PMC13055268; doi:10.1128/spectrum.03167-25)
Supplement: Table S2 — Primers used in this study. [file spectrum.03167-25-s0009.pdf]

Table S2 Primers used in this study

Primer	Sequence (5'→3')	Purpose
NagPN-F<br>NagPN-R	GCGTCTAGAGGGGCGGAGTCTGTAAC<br>GCACTGCAGCGATGGCGATGGAGGT	pΔNagP construction
NagPC-F<br>NagPC-R	CCTGTCTGCAGTGTTCTATTCGGTGATC<br>CCTCGGCAAGCTTCAGGGTGGACGTG	
NagB-F<br>NagB-R	TGCTCTAGATGGTTGTGCGTGGTT<br>CGCGAGCTCATCGCGGAAGTCGT	pΔNagB construction
NagA-F<br>NagA-R	GCAAAGCTTCTACCGCGCTATCAATGC<br>CAGGAATTCGGTGTATCCCACCAG	pΔNagA construction
NagFN-F<br>NagFN-R	CGGAAGCTTCGTTGATGGCGTTCAC<br>GTCGCATGGCTTGTTGACCAGCAGCATC	pΔNagF construction
NagFC-F<br>NagFC-R	CATGCATGCTGTCGGTGGTGCTGATG<br>TGGTCTAGAAATCAGGAACTGCGTAT	
NagA2N-F<br>NagA2N-R	GGCAAGCTTCAACGTGCTGCTGACC<br>TGGTCTAGATGCGAATCGAAGTGG	pΔNagA2 construction
NagA2C-F<br>NagA2C-R	ATGTCTAGAAGGTGTTTCCACCTT<br>GCGGAATTCGCAGTTGCTTCAAGATAGG	
NagKN-F<br>NagKN-R	GCGAAGCTTCGAACAGGACCTGCTGGAA<br>CCGTCTAGAGGAAGCCGTCGTAGTCAC	pΔNagK construction
NagKC-F<br>NagKC-R	GCCTCTAGACCCTCGTTTTCCTGATG<br>GCGGAATTCATGCTGCAGTCGAGATG	
