# Supplementary material for: N-acetylglucosamine utilization and impact on antibiotic susceptibility, oxidative stress tolerance, and swimming in Stenotrophomonas maltophilia
Source: Microbiol Spectr. 2026 Mar 16;14(4):e03167-25. doi: 10.1128/spectrum.03167-25 (PMC13055268; doi:10.1128/spectrum.03167-25)
Supplement: Table S3 — Impact of GlcNAc on swimming motility of KJ, KJΔNagA, and KJΔNagPΔNagF. [file spectrum.03167-25-s0010.pdf]

Table S3 Impact of GlcNAc on swimming motility of KJ, KJΔNagA, and KJΔNagPΔNagF

Strain	Swimming zone (mm)	
	GlcNAc(-)	GlcNAc(+)
**KJ**	34 ± 2.0	40 ± 2.0
**KJΔNagA**	29 ± 2.8	30 ± 2.2
**KJΔNagPΔNagF**	26 ± 1.9	28 ± 2.0
